# Investigating the use of machine learning to generate ventilation images from CT scans

**DOI:** 10.1002/mp.15688

**Published:** 2022-05-15

**Authors:** James Grover, Hilary L. Byrne, Yu Sun, John Kipritidis, Paul Keall

**Affiliations:** ^1^ ACRF Image X Institute Faculty of Medicine and Health The University of Sydney Sydney Australia; ^2^ School of Physics The University of Sydney Sydney Australia; ^3^ Northern Sydney Cancer Centre Royal North Shore Hospital St Leonards Australia

**Keywords:** functional lung imaging, Galligas PET, machine learning, ventilation

## Abstract

**Background:**

Radiotherapy treatment planning incorporating ventilation imaging can reduce the incidence of radiation‐induced lung injury. The gold‐standard of ventilation imaging, using nuclear medicine, has limitations with respect to availability and cost.

**Purpose:**

An alternative type of ventilation imaging to nuclear medicine uses 4DCT (or breath‐hold CT [BHCT] pair) with deformable image registration (DIR) and a ventilation metric to produce a CT ventilation image (CTVI). The purpose of this study is to investigate the application of machine learning as an alternative to DIR‐based methods when producing CTVIs.

**Methods:**

A patient dataset of 15 inhale and exhale BHCTs and Galligas PET ventilation images were used to train and test a 2D U‐Net style convolutional neural network. The neural network established relationships between axial input BHCT image pairs and axial labeled Galligas PET images and was evaluated using eightfold cross‐validation. Once trained, the neural network could produce a CTVI from an input BHCT image pair. The CTVIs produced by the neural network were qualitatively assessed visually and quantitatively compared to a Galligas PET ventilation image using a Spearman correlation and Dice similarity coefficient (DSC). The DSC measured the spatial overlap between three segmented equal lung volumes by ventilation (high, medium, and low functioning lung [LFL]).

**Results:**

The mean Spearman correlation between the CTVIs and the Galligas PET ventilation images was 0.58 ± 0.14. The mean DSC over high, medium, and LFL between the CTVIs and Galligas PET ventilation images was 0.55 ± 0.06. Visually, a systematic overprediction of ventilation within the lung was observed in the CTVIs with respect to the Galligas PET ventilation images, with jagged regions of ventilation in the sagittal and coronal planes.

**Conclusions:**

A convolutional neural network was developed that could produce a CTVI from a BHCT image pair, which was then compared with a Galligas PET ventilation image. The performance of this machine learning method was comparable to previous benchmark studies investigating a DIR‐based CTVI, warranting future development, and investigation of applying machine learning to a CTVI.

## 1 INTRODUCTION

Radiation‐induced lung injury (RILI) is a side effect of radiotherapy treatment to the thoracic region taking the form of radiation pneumonitis and radiation fibrosis.^1^ Radiation pneumonitis has a symptomatic and fatal incidence of 29.8% and 1.9%, respectively, dependent on dosimetric factors and tumor location.^2^

Ventilation imaging of the lungs, before radiotherapy, can assist treatment planning in delineating dose coverage of a patient.^3^ RILI shows higher correlation with dose to functional lung (as can be defined by ventilation imaging) than homogenous lung metrics.^4^, ^5^ Accordingly, adapting radiotherapy treatment planning to reduce dose to high functioning lung (HFL) could reduce the incidence of RILI.

There are a variety of ventilation imaging modalities described in the literature, including SPECT, CT, MRI, and PET.^6^, ^7^

SPECT ventilation imaging is generally performed with the inhalation of a radioaerosol (in most cases ^99m^Tc‐Technegas), which is subsequently detected by a SPECT‐CT scanner.^8^ A retrospective study showed that SPECT ventilation imaging is a feasible method to reduce dose to ventilated lung through functionally adapted IMRT treatment planning.^9^

MRI ventilation imaging generally refers to the inhalation of hyperpolarized ^3^He gas followed by a thoracic MR imaging sequence. In the literature, it has been described as a single breath‐hold of hyperpolarized ^3^He gas mixture.^10^ Hyperpolarized helium MR ventilation imaging has been investigated in the literature for functional lung avoidance in radiotherapy.^11^ MR ventilation imaging has been used in conjunction with CT ventilation imaging (CTVI) to quantify cross‐modality correlation and similarity.^12^, ^13^

PET ventilation imaging follows a similar acquisition protocol to SPECT ventilation imaging with the main exception being the radioaerosol and nuclear medicine detector. The radioaerosol typically used in PET ventilation imaging is Galligas PET, which is used as a reference modality in this study. The differences of PET ventilation imaging compared to SPECT ventilation imaging have been described in the literature with PET offering improved spatial resolution and sensitivity at the limitation of availability.^14^ Galligas PET ventilation images have been used a reference modality for a variety of studies quantifying the cross‐modality correlation and accuracy of CTVIs.^15^, ^16^, ^17^, ^18^

CT ventilation imaging generally refers to when a treatment planning 4DCT is used to produce an estimation of ventilation within the lungs, thus producing a CTVI. An alternative CT‐based ventilation imaging modality uses xenon‐enhanced CT (XeCT), where a patient inhales an Xe–O_2_ gas while undertaking a breath‐hold CT (BHCT).^19^ Conventional CTVIs rely on deformable image registration (DIR) to the inhalation and exhalation respiratory phases of a 4DCT then the application of a ventilation metric to estimate ventilation (in this paper, these will be referred to as “DIR‐based”). The benefit of a CTVI is that the CT images are typically available from treatment planning, reducing the clinical time and monetary costs associated with nuclear medicine ventilation imaging. As DIR‐based CTVIs rely on DIR and the selection of a ventilation metric, these images are sensitive to choice of DIR technique, choice of ventilation metric, and inter‐patient variability. This results in a large variability in CTVIs and their correlation with SPECT, PET, and XeCT‐based ventilation images.^18^ Replacing a regular 10‐phase 4DCT with BHCT has improved the cross‐correlation of the produced CTVIs with a Galligas PET reference modality.^15^ Substituting DIR with a direct estimation of ventilation using the 4D CT Hounsfield unit (HU) values has improved the cross‐correlation of the produced CTVIs with a Galligas PET reference modality.^16^ A large systematic review found that further work is warranted in standardizing CTVI methodologies and comparisons with reference modalities.^7^

Machine learning has revolutionized the medical physics world and is an emerging field of research. One type of machine learning is deep learning and is characterized by hidden layers and feature learning. A recent systematic review has found that deep learning applied to functional lung imaging is a relatively small field with good opportunities for further research.^20^ 4DCT perfusion images have been synthesized using a deep learning approach with MAA‐SPECT as the nuclear medicine ground‐truth.^21^ Deep learning has been implemented to generate CTVIs from 4DCT with DIR‐based CTVIs as the reference ground‐truth.^22^ Deep learning has also been successfully used to produce CTVIs from 4DCT with Technegas SPECT as the nuclear medicine ground‐truth, which showed a higher degree of correlation compared with DIR‐based CTVIs.^8^

In this study, we generated CTVIs using deep learning with Galligas PET ventilation images as the nuclear medicine ground‐truth. The significance of using Galligas PET as a reference imaging modality as opposed to Technegas SPECT is based upon Galligas PET's increased resolution and sensitivity.^14^ In utilizing a higher resolution label in a deep learning framework, the synthesized CTVIs will learn from higher resolution labels and will attempt to predict higher resolution ventilation images. In a systematic review of functional lung imaging in radiotherapy, it was recommended ventilation images with high spatial resolution be used for functionally adapted radiotherapy treatment planning.^6^ These CTVIs were then compared to Galligas PET ventilation images to assess the deep learning model's performance.

## 2 MATERIALS AND METHODS

**FIGURE 1:**
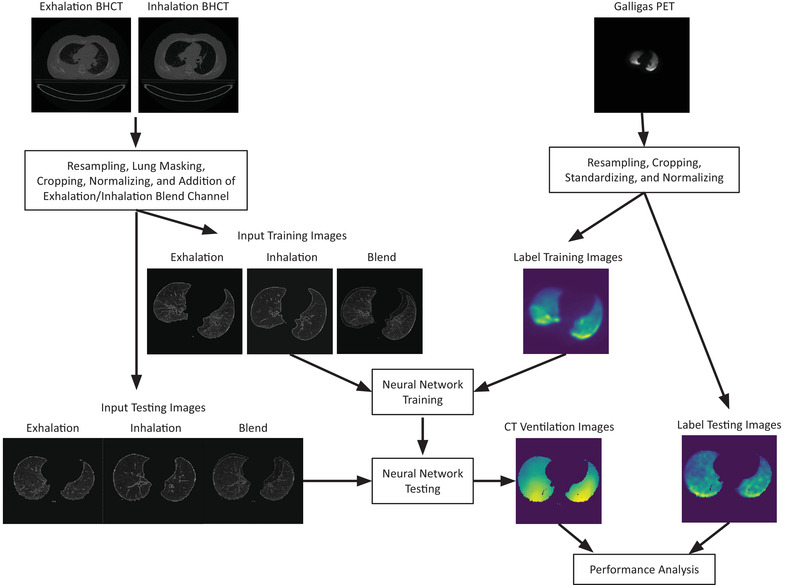
Overview of the methodology. Starting at the top is the acquired BHCT image pairs and Galligas PET ventilation images. These images were preprocessed and split into training and testing groups based on eightfold cross‐validation. The neural network used input preprocessed BHCT and labeled preprocessed Galligas PET images to train. Once trained, the testing preprocessed BHCT images were used by the neural network to make CT ventilation images. These CTVIs were then qualitatively and quantitatively compared to the testing labeled preprocessed Galligas PET images. BHCT, breath‐hold CT; CTVIs, CT ventilation images

An overview of the entire methodology of this study is presented in Figure 1.

### 2.1 Patient data acquisition

The patient images used in this study were acquired at Royal North Shore Hospital between 2013 and 2015 for a previous study investigating CTVI using 4DCT and BHCT pairs, which had the Health District Ethics Committee's approval (HREC/12/169) and was registered to the Australian New Zealand Clinical Trials Registry (ACTRN12612000775819).^15^ A total of 18 lung cancer patients were enrolled with varying ages (54–73 years), lung tumor staging (II–IV), and chronic pulmonary disease (mild to severe). A total of 16 BHCT pairs and corresponding Galligas PET image sets were successfully acquired in one imaging session using a Siemens Biograph mCT.S/64 PET/CT scanner (Siemens, Knoxville, USA). Data from 15 of these patients were available for this study due to a duplication error.

Acquisition parameters for the BHCT image pairs are described as follows. Patients were instructed, using audiovisual biofeedback, to hold their breath at 80% peak inhale and 80% peak exhale for 10 s each. Scan parameters of the BHCT pairs were 120 kVp, 120 mA s, and a pitch of 0.8 producing a field size of approximately 50 cm of the thoracic region of the patient. These reconstructed BHCT image pairs had a voxel size of 0.96 mm × 0.96 mm × 1.8 mm (*x*, *y*, *z*) and units of HUs. Each patient had around 170 slices, each consisting of 512 × 512 pixels.

Acquisition parameters for the Galligas PET images are described as follows. Patients inhaled an estimated activity of 20 MBq of Galligas radiotracer. The following acquisitions were performed under free‐breathing with the patient imaged at two bed positions for 5 min each. The attenuation correction low‐dose CT had scan parameters of 120 kVp, 50 mA s, and a pitch of 0.8. The reconstructed Galligas PET images had a voxel size of 2.04 mm × 2.04 mm × 2.2 mm (*x*, *y*, *z*) and units of kBq/mL. Each patient had around 160 slices and each contained 400 × 400 pixels.

### 2.2 Image preprocessing

The pipeline from acquired patient images to the neural network is illustrated in Figure 1. The BHCT image pairs and Galligas PET images were both resampled, using B‐spline interpolation, into a voxel size of 1 mm × 1 mm × 1 mm (*x*, *y*, *z*) using SimpleITK on Python.^23^ Resampling was performed to ensure a voxelwise correspondence between the BHCT image pairs and Galligas PET images. A 1‐mm^3^ voxel size was selected not to lose the relatively high spatial resolution afforded by the BHCT pairs that increased the Galligas PET image resolution. As the Galligas PET images were acquired in the same imaging session as the BHCT pairs, rigid shifting using visual inspection was used to align the two modalities, with the goal to align the Galligas PET image to the exhalation phase of the BHCT image pair.

The BHCT image pairs were lung masked by a −500‐HU intensity threshold filter, followed by a patient‐dependent combination of erosion, dilation, and flood‐filling morphological operations using scikit‐image on Python.^24^ Lung masking was applied to the union of exhalation and inhalation phases of the BHCT images. Both the BHCT image pairs and Galligas PET images were cropped into 250‐axial slices, each containing 304 × 304 pixels. These dimensions were sufficient to encompass the lung for each patient.

The BHCT image pairs were both min–max normalized to 0 and 1. The Galligas PET images contained a large range of radioactivity values caused by small bright spots of radioaerosol clumping. Consequently, when performing a min–max normalization in isolation, most voxels were assigned a near‐zero radioactivity value. As a result, an additional method of standardization was applied. This standardization calculates the mean (*μ*) and standard deviation (*σ*) radioactivity of the voxels contained within the patient's lung. Any voxel with a radioactivity value greater than *μ* + 4*σ* is set equal to *μ* + 4*σ*. This method of standardization is consistent with a clinical implementation of Galligas PET images in radiotherapy treatment planning.^25^ After standardization, the Galligas PET images were min–max normalized to 0 and 1. The normalization and standardization were performed globally across the entire patient lung volume.

### 2.3 Machine learning development

A 2D U‐Net style convolutional neural network was used to train and produce the axial ventilation images which, when assembled, provided a 3D ventilation map of the patient's lung. A 2D framework was developed due to computational limitations with the axial plane being used as the acquired images had the highest resolution in the axial plane. The framework for developing the neural network was TensorFlow 2.6.0.^26^ The input images were three channels consisting of the exhalation, inhalation, and average images of exhalation/inhalation BHCT images. A third channel was necessitated by the image augmentation framework used. The addition of the third average image channel was anticipated to assist in training lung boundaries and shared anatomy between exhalation and inhalation. Compared with using a blank (i.e., all zeros) third channel, this average image produced an approximate 2% increase in the mean Spearman correlation over the entire patient dataset.

Eightfold cross‐validation was used to measure the robustness and increase the validity of the results attained by the neural network. In the case of 15 patients, a patient was used for testing twice thus ensuring that each fold used 13 training patients and 2 testing patients. Due to the relatively small patient cohort of just 15 patients, augmentation was used to reduce overfitting in the neural network. The specific augmentation used was up to 10‐degree axial rotation and up to 20‐pixel translation of the images (in the anterior–posterior and left–right direction).

**FIGURE 2:**
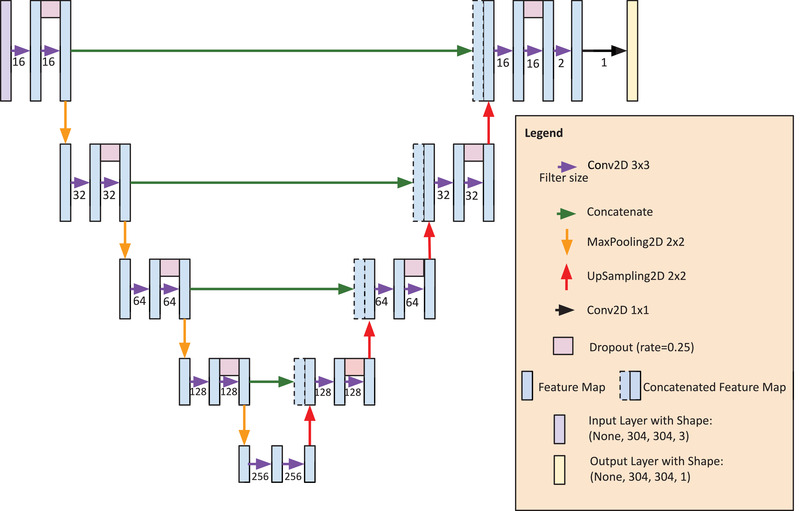
The neural network architecture. Starting at the top left and finishing at the top right is the path each augmented input image takes as the machine learning algorithm learns. The total number of trainable parameters in this model is 1962 901 with 0 non‐trainable parameters. The input shape contains three channels corresponding to exhalation, inhalation, and alpha blend BHCT images. The output shape contains only one channel corresponding to a predicted Galligas PET image. Convolutional layers used ReLU activation with He kernel initialization and the “same” padding except for the final output layer, which used sigmoid activation. The pooling layers had a stride size of two. BHCT, breath‐hold CT

For each cross‐validation fold, the 13 training patients’ axial images were contained within one stack comprising a 3‐channel input BHCT (exhalation, inhalation, and average image of exhalation/inhalation) and a 1‐channel‐labeled Galligas PET image. A random 10% of this training stack was used for validation during training, to track the loss and accuracy of the neural network during training. The architecture of the neural network was based on U‐Net following a similar architecture proposed by Liu et al.^8^, ^27^ This architecture contains a contracting downsampling path followed by an expanding upsampling path with concatenations between each path for feature preservation. The architecture of the neural network is outlined in Figure 2.

Standard regression loss functions (such as mean squared error and mean absolute error) for training the neural network produced uniform blank images, hypothesized to be caused by the large proportion of blank voxels in the dataset (i.e., area outside the patient lung). Accordingly, a loss function that penalized underpredictions far more than overpredictions was used. The type used in this neural network was focal loss, which is a modified form of binary cross‐entropy, with two tuneable parameters *α* and *γ*.^28^ After performing a trial‐and‐error method, the final values for the parameters of *α* and *γ* were 1.5 and 5.0, respectively. The model was trained for 15 epochs for each cross‐validation fold using an Adam optimizer and a learning rate of 0.0003. Training time was approximately 7.5 h per cross‐validation fold using an Intel Core i7‐1065G7 CPU. The produced CTVIs were normalized between 0 and 1 globally across the entire 3D CT ventilation map. Each axial slice for each patient was saved as an image stack that provided a full 3D ventilation map of the patient's lungs, which could be viewed in axial, coronal, and sagittal planes. A full ventilation map, consisting of 250 axial slices, took approximately 10 s to produce using an Intel Core i7‐1065G7 CPU.

### 2.4 Performance evaluation

Qualitative analysis was performed visually, by comparing the CTVIs to the Galligas PET images. Two quantitative performance metrics were selected for this study based on their prevalence in other research concerning CTVI: the Spearman correlation and the Dice similarity coefficient (DSC). To ensure that these performance metrics were performed on the voxel values contained only within the patient lung, a lung mask of the exhalation phase of the BHCT images was used.

The Spearman correlation is used to describe the monotonicity of two datasets and takes a value between −1 (perfect negative correlation) and +1 (perfect positive correlation).

The DSC is used to describe the spatial overlap between structures and takes a value between 0 (no spatial overlap) and 1 (full spatial overlap). DSC can be expressed in terms of true positive (TP), false positive (FP), and false negative (FN):

$$\mathrm{DSC}=\frac{2\mathrm{TP}}{2\mathrm{TP}+\mathrm{FP}+\mathrm{FN}}$$

Three functional volumes were defined in this study, in‐line with the previous work applying deep learning to CTVI.^8^ The lung volume, as defined by the exhalation lung mask, was split into three equal volumes based on voxel values (i.e., ventilation). The process of determining these three equal volumes involves ordering the voxel values by number and determining which two values (first and second threshold values) split this ordered set of voxel values into three equal parts. These three functional sub‐volumes corresponded to HFL, medium functioning lung (MFL), and low functioning lung (LFL) as defined by the ventilation. These functional sub‐volumes were computed for the CTVIs and Galligas PET images, and a DSC was computed for each sub‐volume. An average value for the DSC over HFL, MFL, and LFL was also calculated to describe the overall spatial overlap between the CTVI and Galligas PET ventilation images.

## 3 RESULTS

**FIGURE 3:**
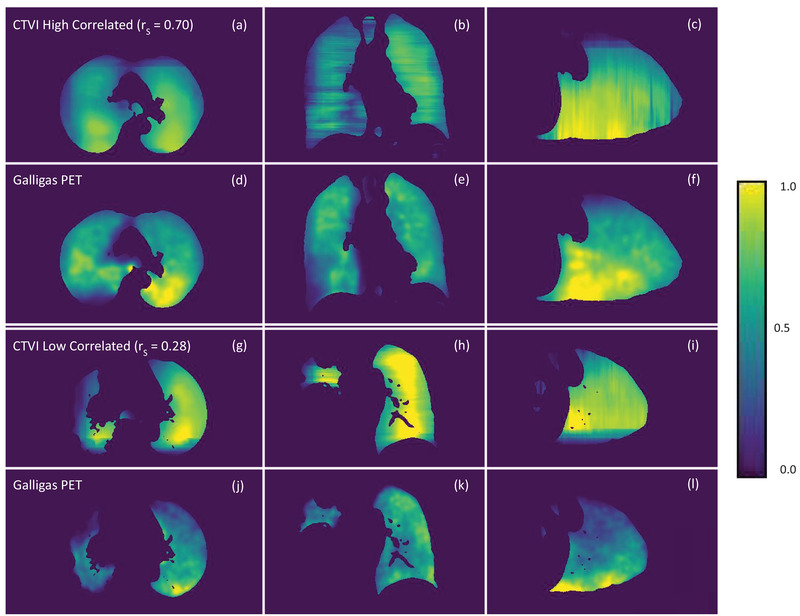
Comparison between neural network produced CTVIs and Galligas PET ventilation images. Images (a)–(c) and the axial, coronal, and sagittal views of a high correlated (Spearman correlation (*r*_S_) = 0.70) CTVI produced by the neural network. Images (d)–(f) are the same axial, coronal, and sagittal views of this same high correlated patient's Galligas PET ventilation image. Images (g)–(i) are the axial, coronal, and sagittal views of a low correlated (Spearman correlation = 0.28) CTVI. Images (j)–(l) are the same axial, coronal, and sagittal views of this same low correlated patient's Galligas PET ventilation image. CTVIs, CT ventilation images

A high and low correlated CTVI (highest/lowest Spearman correlation) is presented in Figure 3 with corresponding Galligas PET ventilation images that, for the purposes of this study, were considered a ground‐truth.

### 3.1 Qualitative performance

The CTVI produced by the neural network was first compared to the Galligas PET images through visual inspection. Even without using filtering as a post‐processing operation, each axial CTVI slice presented a smoothness among regions of low, medium, and high ventilation. This smoothness resulted in a difficulty in predicting small pockets of high and low ventilation within the patient lung. When displaying the ventilation maps in the coronal or sagittal plane, the ventilation showed distinct jagged edges in the superior–inferior (SI) direction.

**FIGURE 4:**
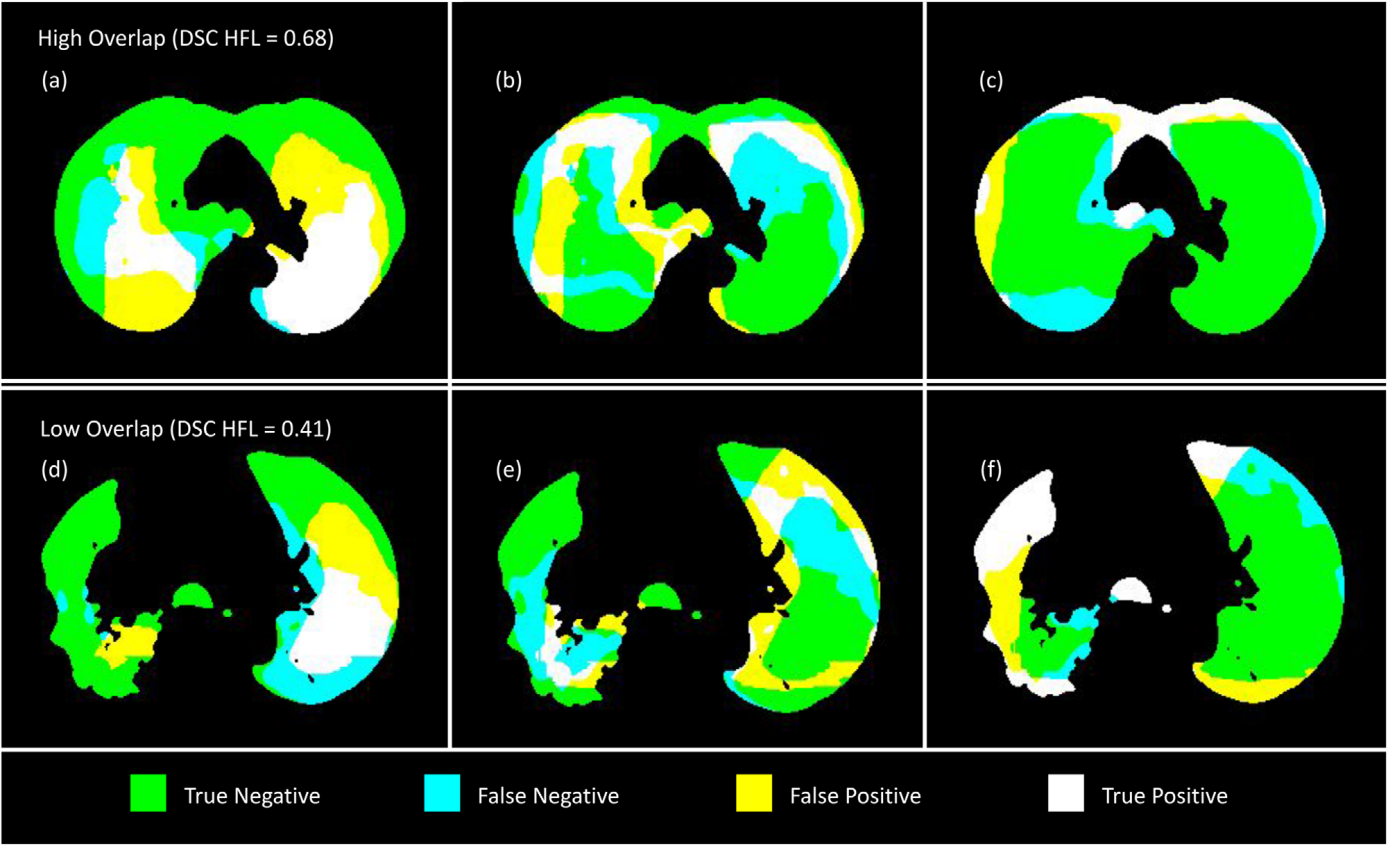
Functional lung regions as defined by the neural network produced CTVI and Galligas PET ventilation image displayed in terms of true/false negative/positive. Images (a)–(c) are the spatial overlap of HFL, MFL, and LFL, respectively, for a CTVI with high spatial overlap (DSC HFL = 0.68). Images (d)–(f) are the spatial overlap of the HFL, MFL, and LFL, respectively, for a CTVI with low spatial overlap (DSC HFL = 0.41). CTVI, CT ventilation images, DSC, Dice similarity coefficient; HFL, high functioning lung; LFL, low functioning lung; MFL, medium functioning lung

The CTVIs tended to show a systematic overprediction of ventilation within the patient lung when compared to the Galligas PET ventilation images. Qualitatively, the HFL was mainly localized around the center and anterior of the patient's lung for both the CTVI and Galligas PET images. The HFL, MFL, and LFL for CTVIs with a high‐and‐low spatial overlap of the HFL are presented in Figure 4. The MFL had the least spatial overlap between the CTVI and the Galligas PET images. Most of the MFLs were defined in regions between the extremities and center of the patient lung. The LFL was confined to mainly the extremities of the lungs in both the CTVIs and the Galligas PET images.

### 3.2 Quantitative performance

**FIGURE 5:**
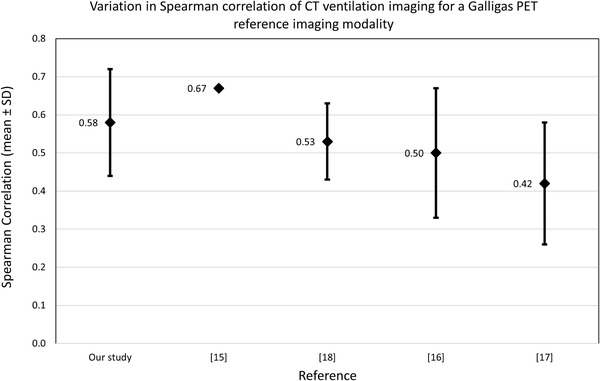
Variation in the Spearman correlation of CT ventilation images to reference Galligas PET images for a variety of studies. The number and diamond refer to the mean value across the testing CT ventilation images (for the best performing algorithm, where applicable). The error bars refer to the standard deviation if provided by the study

Each testing patient across the cross‐validation was masked using an exhalation lung mask ensuring that quantitative performance measures were only taken for voxels contained within the lung. For each patient, the Spearman correlation and the DSC for HFL, MFL, and LFL was calculated between their CTVI and their Galligas PET ventilation image. The mean values across the 15 patients are presented in Table 1. Several studies involving various methods of CTVI generation with a reference to a nuclear medicine ground‐truth were compared to our quantitative results and are presented in Figure 5.

**TABLE 1:** Performance measures of the machine learning generated CT ventilation images

**Performance measure**	**Mean value across 15 patients (mean ± standard deviation)**
Spearman correlation	0.58 ± 0.14
DSC high functioning lung	0.61 ± 0.09
DSC medium functioning lung	0.43 ± 0.05
DSC low functioning lung	0.62 ± 0.07
DSC average	0.55 ± 0.06

Abbreviation: DSC, Dice similarity coefficient.

## 4 DISCUSSION

Machine learning was successfully used to generate CTVIs from BHCT image pairs without the need for DIR or ventilation metrics. A neural network was trained and developed on a CPU‐only laptop that could produce a full ventilation map of an unseen patient within 10 s. The pre‐ and post‐processing, training, inference, and performance measures were able to be performed on a CPU‐only laptop, meaning that this methodology is widely accessible especially where hardware limitations are present. Comprehensive performance analysis of the neural network was conducted showing promising results even when using a reasonably small patient dataset with minimal pre‐ and post‐processing.

The mean Spearman correlation between CTVIs and a Galligas PET reference image for a variety of previous studies is presented in Figure 5. The mean Spearman correlation achieved in this study was 0.58 ± 0.14 (standard deviation) ranging from 0.28 to 0.70 for a 15‐patient dataset.

A direct comparison with a conventional method of producing CTVIs using DIR‐based methodology is provided by Eslick et al., as their study used the same BHCT image pairs and Galligas PET images.^15^ The mean Spearman correlation they achieved for 16 patients (15 of which were used in this study) was 0.67, which was higher than the mean achieved in our study and is represented as Ref. [15] in Figure 5 (a standard deviation was not provided in their study).^15^ A lower mean Spearman correlation is observed and is speculated to be caused by the relatively low patient dataset. DIR‐based methods are impervious to small patient datasets and are well suited where there is a lack of patient data.

The AAPM grand challenge VAMPIRE had participants to submit their own CTVIs, based on a provided 4DCT, which were compared for a variety of reference imaging modalities, including Galligas PET. The best performing DIR‐based algorithm using Galligas PET as a reference modality in this grand challenge had a mean Spearman correlation of 0.53 ± 0.10 (standard deviation), represented as Ref. [18] in Figure 5.^18^ A study investigating the use of a non‐DIR‐based method of producing CTVIs with a Galligas PET reference modality observed a mean Spearman correlation of 0.50 ± 0.17, represented as Ref. [16] in Figure 5.^16^ The mean Spearman correlation for a study correlating DIR‐based CTVIs with a Galligas PET reference image for 12 patients was 0.42 ± 0.16 (standard deviation), represented as Ref. [17] in Figure 5.^17^ There are several fundamental differences in the different studies presented in Figure 5. An example of this is the type of CT used to generate the CTVI, in all cases but our study and Eslick et al., these other studies used 4DCT, instead of BHCT, which can reduce the correlation of the produced CTVIs.^15^ Consequently, Figure 5 is presented in this study as an initial comparison of CTVI methodologies when a Galligas PET reference modality is used.

Although Liu et al. did not use Galligas PET as the reference modality, rather Technegas SPECT, it is still useful to compare another similar deep learning approach to generating CTVIs. The mean Spearman correlation (across a dataset of 50 patients) achieved by Liu et al. who used a similar neural network was 0.73 ± 0.16 (standard deviation).^8^ The CT input images were 10‐ and 2‐phase 4DCT.

A comparison for the DSC between this study and Liu et al. is possible due to the definition of the functional lung being consistent between their study and this study. The mean DSC across the HFL, MFL, and LFL achieved by Liu et al. was 0.73 ± 0.09 (standard deviation).^8^ They similarly observed a reduction in the DSC for MFL in comparison with the HFL and LFL. The mean DSC achieved in this study was 0.55 ± 0.06.

A multitude of factors underpin the varying successes in replicating a nuclear ventilation image by use of CT. A major factor influencing how a machine learning algorithm generalizes in testing is the size of dataset used for training. The patient dataset in our study was relatively small, only containing 15 patients, compared to other studies. The Spearman correlation and DSC would be expected to increase with an increasing patient dataset. A large variety of lung ventilation was observed in the Galligas PET patient dataset, which is a result of patient characteristics (lung tumor staging, chronic obstructive pulmonary disorder, and age) and can be observed qualitatively (uniformity of ventilation) and quantitatively (maximum/mean measured ventilation). Figure 3 shows a particularly low correlated CTVI (Spearman correlation = 0.28) with reference to the Galligas PET ventilation image. Noticeably the sparse pockets of ventilation were unable to be replicated in the neural network produced CTVI.

Due to computational limits, a 3D neural network was not employed in our study. It is expected that a 3D neural network would increase the Spearman correlation and DSC as the model would be able to learn from a full patient volume instead of individual slices as shown in our study through the coronal and sagittal views of the CTVIs, which could also help remove the distinct jagged edges in the SI direction, as shown in Figure 3.

Our study warrants the future application and study into using machine learning to generate CTVIs. With larger patient datasets and more sophisticated machine learning algorithms, CTVIs derived using machine learning can expect to see higher cross‐modality correlation with reference modalities. This can have the direct benefit in radiotherapy treatment planning, improving outcomes for patients and their families.

## 5 CONCLUSIONS

A machine learning algorithm was developed in the form of a neural network that could produce a CTVI without the use of DIR or ventilation metrics within 10 s on a laptop. Using a small patient dataset with a large variance in lung ventilation, the mean Spearman correlation was 0.58 ± 0.14, and the mean DSC over low, medium, and HFL was 0.55 ± 0.06. These performance measures are comparable with conventional DIR‐based methods warranting the further investigation into applying machine learning to CT ventilation imaging.

## CONFLICT OF INTEREST

Paul Keall is an inventor on an unlicensed patent related to CT ventilation owned by Virginia Commonwealth University. John Kipritidis and Paul Keall are authors of CT Ventilation software that has been recorded as intellectual property by the University of Sydney.

## Data Availability

The authors are not able to share data at this time.
